# Elective Tracheostomy in Critically Ill Children: A 10-Year Single-Center Experience From a Lower-Middle Income Country

**DOI:** 10.7759/cureus.9080

**Published:** 2020-07-09

**Authors:** Sidra Ishaque, Anwar Haque, Saqib H Qazi, Hamdan Mallick, Saad Nasir

**Affiliations:** 1 Pediatrics, The Aga Khan University Hospital, Karachi, PAK; 2 Pediatrics, The Indus Hospital, Karachi, PAK; 3 Pediatric Surgery, The Aga Khan University, Karachi, PAK; 4 Medicine, The Aga Khan University Hospital, Karachi, PAK; 5 Internal Medicine, United Medical and Dental College, Creek General Hospital, Karachi, PAK

**Keywords:** tracheostomy, critically ill children, prolonged ventilation, outcomes, timing, elective

## Abstract

Objective

Tracheostomy is a commonly performed procedure amongst critically ill patients, especially in cases of prolonged mechanical ventilation (PMV). This study aimed to describe the indications, clinical characteristics, and outcomes of elective pediatric tracheostomies in critically ill children at our center.

Methods

A retrospective review of medical records of children who underwent elective tracheostomies in our pediatric intensive care unit (PICU) was conducted from January 2009 to June 2018. Data were extracted based on demographics, indications of tracheostomy, and patient outcomes. Results were reported as mean with standard deviation and as frequencies with percentage.

Results

Of the 3,200 patients admitted to the PICU during the study period, 1,130 were intubated. A total of 48 (4.2% of 1,130) children underwent an elective tracheostomy. 30/48 (62.5%) children had an early tracheostomy. 34/48 (71%) patients were males. Approximately 25% of our patients undergoing a tracheostomy had an underlying neurological condition as the primary diagnosis, followed by respiratory conditions (23%). The most common indications for elective tracheostomy were PMV (>7 days) (n=24, 50%) and extubation failure (n=9, 18.7%). Early tracheostomy (<14 days) had better patient outcomes in terms of ventilator-free days (8.57±4.64 in early tracheostomy vs. 6.38±6.17 days in late tracheostomy, P=0.04). The sedation-free days and ICU-free days were also significantly increased in the early tracheostomy group than in the late tracheostomy group. The successful weaning and ICU discharge rate were significantly higher in the early tracheostomy group than in the late tracheostomy group (78.1% vs. 59.7%, P<0.05; and 69.2% vs. 49.5%, P<0.05, respectively). Ventilator-associated pneumonia was more common in the late tracheostomy group (n= 14, 77%), compared to early tracheostomy group (n=12, 40%) (P=0.03). Two patients expired from tracheostomy-related complications.

Conclusion

PMV was the most common indication for an elective tracheostomy. Early tracheostomy is associated with improved patient outcomes; therefore, a standardized approach toward mechanically ventilated children is recommended.

## Introduction

Hippocrates described tracheostomy as “*making a hole in the airway*” to relieve upper airway obstruction [1]. In 1766, Carel performed the first successful emergent tracheostomy in a child to bypass the airway obstruction from a bean, and for a very long-time, airway obstruction was the only indication for tracheostomy [2]. In patients with acute respiratory failure who require prolonged mechanical ventilation (PMV), tracheostomy has become an alternative method to endotracheal intubation. This is because prolonged endotracheal intubation is associated with a high risk of developing ventilator-associated pneumonia, while tracheostomy is a safer procedure, associated with decreased ventilator-associated morbidity and mortality [3-5]. Tracheostomy has become an important option for an earlier transition of children from the pediatric intensive care unit (PICU), allowing earlier discharge. The advantages of an early elective tracheostomy in the PICU include improved patient comfort, early weaning from mechanical ventilation, better management of pulmonary toilet, and lesser need for sedative drugs. All these factors lead to a lesser cost of care, decreasing the financial burden on the families [6]. The indications, techniques, and timing for a tracheostomy in critically ill children are debatable. Various studies report PMV as the most common indication for an elective tracheostomy [7-9]. However, Schweiger et al., in their review of tracheostomy indications at their center, reported a large proportion (83%) of patients undergoing tracheostomy because of an upper airway obstruction [10]. Most studies concerning tracheostomy in critically ill children are retrospective reviews from single centers, involving few children, making it a tough area to extrapolate decisions regarding patient management. There is a dearth of literature regarding elective tracheostomies in critically ill children from lower-middle income countries to describe the uses and outcomes of tracheostomy in improving patient comfort in children needing PMV. This is the first detailed study spanning over 10 years from Pakistan. Through this study, we aim to help physicians understand the significance of an earlier tracheostomy in critically ill children by focusing on the timing and outcomes of tracheostomy.

## Materials and methods

We conducted a retrospective analysis of all children (aged one month to 16 years) undergoing elective tracheostomy from January 2009 to June 2018 at the PICU of The Aga Khan University Hospital, Karachi, Pakistan. Institutional Review Board (IRB) approval was sought from the hospital’s ethical review committee. Informed consent was obtained from the parents and family with regards to the procedure. Patients with an existing tracheostomy or who underwent an emergent tracheostomy placement were excluded. Decision making for tracheostomy was performed on a case-by-case basis and was made in an interdisciplinary fashion including the critical care team, otolaryngology service/pediatric surgery service, and patient caregivers.

Patients were stratified by the number of days of mechanical ventilation elapsed before tracheostomy based on previously published definitions [11]. Early tracheostomy (ET) was defined as procedures done less than 14 days following mechanical ventilation and late tracheostomy (LT) as greater than or equal to 14 days following mechanical ventilation. PMV was defined as a patient requiring more than seven days of ventilatory support as per the institutional criteria. Extubation failure was defined as an inability to sustain spontaneous breathing after removal of the artificial airway, an endotracheal tube or tracheostomy tube (TT), and a need for reintubation within a specified period: either within 24-72 hours or up to seven days. Early extubation failure was defined as the need for reintubation less than 48 hours after the first extubation. This will mean a failed first extubation attempt within the first 48 hours. Late extubation failure was defined as the need for reintubation in the period between after 48 hours since the first extubation. ICU-free days, estimated based on definitions previously defined as the number of days a patient spends out of the ICU from the day of ICU admission to 28 days thereafter [12].

Patients going home with a TT were given instructions and training regarding care and changing of TT in the hospital. They were provided with a spare TT, suction catheters, and a suction machine. Patients were discharged only when the caregiver and physician were comfortable that the degree of training required for the management of the tube was adequate.

Data collection and analysis

We collected data through a study questionnaire using inpatient medical records by two independent reviewers with a similar protocol as described in a previously published study from our center [13]. Data collection included demographic details (age, gender, weight, height) as well as clinical details including diagnostic categories, indications for tracheostomy, the day of admission when tracheostomy was done, Pediatric Risk of Mortality (PRISM III) score, and outcome data (duration of mechanical ventilation, hospital length of stay [LOS], intensive care unit LOS, and complications of tracheostomy) [14]. Data were entered and analyzed using Statistical Package for Social Science (SPSS) Version 21 (IBM Corp., Armonk, NY) to calculate descriptive statistics such as mean and standard deviation for continuous variables and frequency and percentage for categorical variables.

## Results

Of a total of 3,200 patients admitted to our PICU over 10 years (2009-2018), 1,130 underwent intubation. Forty-eight (4.2% of 1,130 patients) elective tracheostomies were performed. 30/48 (62.5%) patients underwent an ET, i.e, within 14 days of mechanical ventilation.

Two patients were excluded because of missing records after discharge from the PICU. A total of 19 patients (40%) had their tracheostomy performed at less than one year of age, followed by 14 children (29%) undergoing tracheostomy between 11 and 16 years. The median age of tracheostomy was eight months, with age ranging from 20 days to 17 years. A total of 34 (71.0%) patients were males, and the median PRISM III score was 14 (interquartile range: 8). Major diagnostic categories at admission included central nervous system diseases (n=12, 25%), respiratory conditions (n=11, 22.9%), sepsis/multiorgan dysfunction syndrome (MODS) (n=8, 16.7%), cardiac diseases (n=6, 12.5%), and miscellaneous conditions (n=11, 22.9%). Among patients with neurological conditions, traumatic brain injury (n=5) was most common followed by meningitis/encephalitis (n=4), Guillain-Barré syndrome (n=2), and neurodegenerative disorders (n=1). Among patients with respiratory conditions, upper airway obstruction (n=6) was most common, followed by complicated pneumonia/acute respiratory distress syndrome (ARDS) (n=3) and foreign body aspiration (n=2). The demographic and diagnostic characteristics of our patients are detailed in Table 1.

**Table 1:** Clinical and demographic features of patients admitted to the pediatric intensive care unit during the study period (n=48) n, sample size; PRISM III, Pediatric Risk of Mortality III; IQR, interquartile range; MODS, multiorgan dysfunction syndrome; ARDS, acute respiratory distress syndrome

Variables	n (%)
Age (years)	
<1 year	19 (40)
1-5 years	8 (17)
6-10 years	7 (16)
11-16 years	14 (29)
Gender	
Male	34 (71)
Female	14 (29)
PRISM III score, median (IQR)	14 (8)
Disease diagnosis categorization at admission	
Central nervous system	12 (25)
Traumatic brain injury	5 (41.6)
Meningitis/encephalitis	2 (16.6)
Guillain-Barre syndrome	4 (33.3)
Neurodegenerative disorders	1 (8.3)
Cardiovascular system	6 (12.5)
Respiratory system	11 (22.9)
Upper airway obstruction	6 (54.5)
Foreign body	2 (18.1)
Complicated pneumonia/ARDS	3 (27.2)
Infection/sepsis/MODS	8 (16.7)
Miscellaneous	11 (22.9)
Total	48

**Figure 1:**
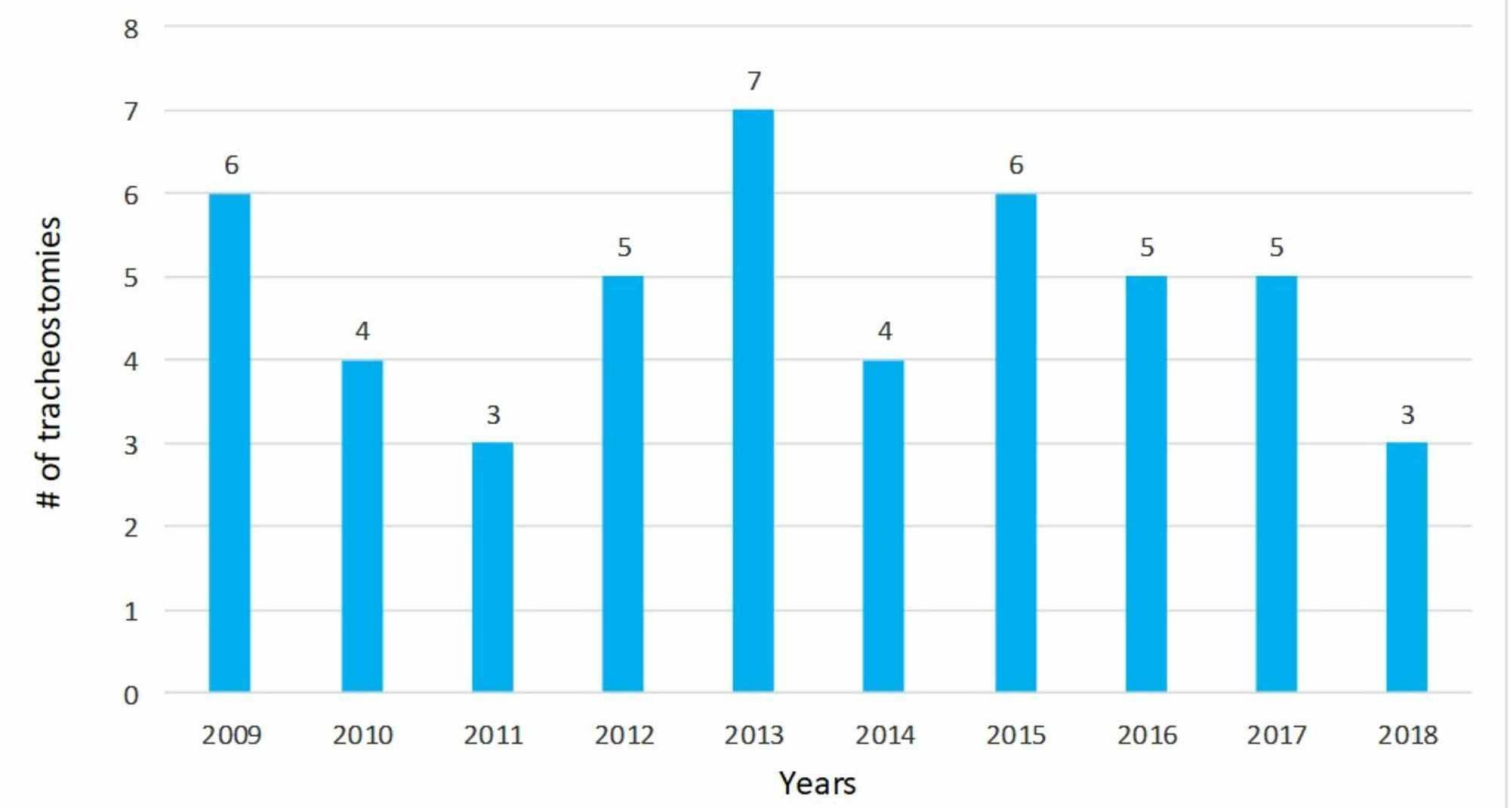
Year-wise trend of the number of tracheostomies performed

The most common indications for elective tracheostomy (Table 2) were PMV (n=24, 50%) and extubation failure (n=9, 18.7%). The number of procedures per year remained stable over the study period (Figure 1)

**Table 2:** Principal indications for tracheostomy placement n, sample size

Indications	n (%)
Prolonged mechanical ventilation	24 (50)
Extubation failure	9 (18.7)
Upper airway obstruction	6 (12.5)
Craniofacial trauma	5 (10.4)
Tracheobronchial toileting	4 (8.3)
Total	48 (100)

Tracheostomy was performed within the first 14 days of mechanical ventilation in 30 (62.5%) of our patients. When compared with those who underwent LT, ET was shown to have better patient outcomes in terms of ventilator-free days (8.57±4.64 in ET vs. 6.38±6.17 days in LT, P=0.04). The sedation-free days and intensive care unit-free days were also significantly increased in the ET group than in the LT group. The successful weaning and intensive care unit discharge rate was significantly higher in the early ET group than in the LT group (78.1% vs. 59.7%, P<0.05; and 69.2% vs. 49.5%, P<0.05, respectively). Ventilator-associated pneumonia was observed in 12 patients (40%) in the ET group and 14 patients (77.7%) in the LT group (P=0.03) (Table 3). The first tracheostomy change happened within the first week to 10 days, depending on the surgeon’s discretion.

**Table 3:** Outcomes amongst patients with early tracheostomy versus late tracheostomy ET, early tracheostomy; LT, late tracheostomy; n, sample size; SD, standard deviation; PICU, pediatric intensive care unit; VAP, ventilator-assisted pneumonia; CI, confidence interval *P-value <0.05

Outcomes	ET (n=30)	LT (n=18)	P-value
Ventilator-free days (mean±SD)	(8.57±4.64)	(6.38±6.17)	0.04*
Length of PICU stay (mean±SD)	(18.45±2.55)	(21.50±3.65)	<0.01*
VAP incidence (n, %, 95% CI)	12 (40%) (15-60)	14 (77.7%) (60-92)	0.03*
Mortality during hospital stay (n, %, 95% CI)	01 (33%) (12-50)	01 (55%) (35-90)	0.66

There were no intraoperative adverse events. A total of 17 children (35%) had complications before the first tracheostomy change. The two early major complications include tube blockage due to thickened secretions (6, 12.5%), and accidental decannulation was seen (2, 4.1%). Two patients expired, both from life-threatening hemorrhage, what was most likely to be trachea-innominate fistula, making it an overall mortality rate of 4%. The first patient was a six-year-old who had undergone repair for a cardiac defect and needed a tracheostomy due to extubation failure. The other patient needed the tracheostomy due to severe craniofacial trauma and the inability to be weaned off the ventilatory support. They developed the complications at days 8 and 10 of tracheostomy, making the first two weeks after tracheostomy critical with regards to patient management.

**Figure 2:**
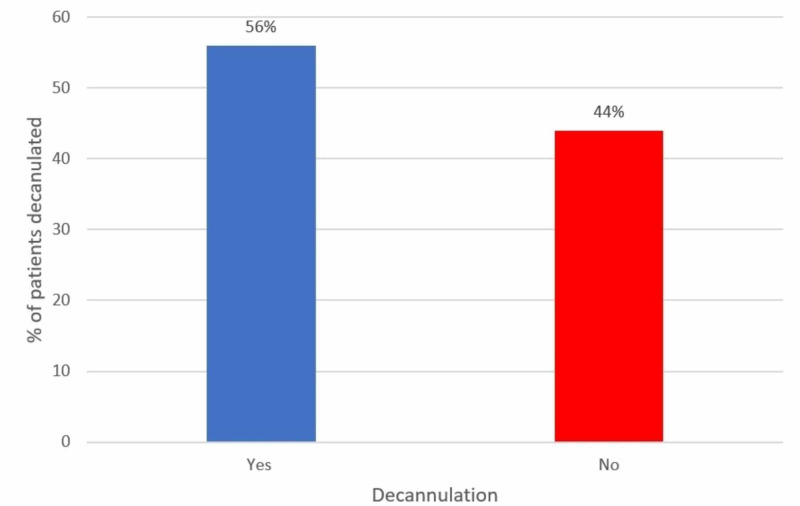
Percentage of decannulation in patients undergoing a tracheostomy

The overall decannulation rate was 56% during the study period (Figure 2).

## Discussion

Tracheostomy refers to making a surgical opening in the trachea, and in critically ill patients, it maintains patency of the airway. It is a common procedure in adults that ranges from 10% to 24% of ventilated patients [15]. From the year 2000 to 2012, 24,354 pediatric tracheostomies were performed in the United States [16]. In a survey done in Canada, the overall rate of pediatric tracheostomy was almost 1.5% of the ventilated patients, similar to ours (1.5%) [17]. Most children in our cohort were under the age of 12 months at the time of tracheostomy placement. These results are consistent with earlier studies in the literature [16,18,19]. The indications for tracheostomy have transformed over the last few decades, reflecting the changes that have occurred in managing critically ill children [20,21]. We now perform tracheostomy in children who need PMV because of respiratory failure, besides those who have upper airway abnormalities or chronic illnesses (neurological impairment, congenital heart, and lung diseases) [15]. Can et al. conducted a retrospective analysis of 63 children undergoing tracheostomy in PICU and showed PMV as the common indication for an elective tracheostomy [22]. Our results are consistent with the data reported in earlier studies [1,19,22]. In our patient population, children who underwent tracheostomy were more likely to have their admission diagnosis grouped as neurological and respiratory (25 vs. 23% of all admissions) and less likely as cardiovascular. The most common diagnoses were traumatic brain injury, followed by upper airway obstruction. PMV because of a respiratory problem was the major indication for an elective tracheostomy in 50% of our population. Extubation failure and upper airway obstruction formed the other 30% of indications. In mechanically ventilated adults, tracheostomy performed during the first two weeks results in favorable outcomes [23,24]. But, no established criteria exist for the timeline for tracheostomy in critically ill children; hence, the decision is at the physicians' discretion based on the clinical profile of the patient. In developed countries such as Canada and the United States, the average time estimated for an elective tracheostomy in children is 21 and 14.4 days, respectively [25]. Puhakka et al. determined an average of 64 days of hospitalization elapsed before tracheotomy was performed in children with an indication [26]. In our series, an overall mean of 18.5 days of mechanical ventilation elapsed before tracheotomy, with 62% of our children undergoing a tracheostomy placement during the first 14 days of mechanical ventilation. The discrepancy in tracheostomy timing between children and adults may be secondary to the heterogeneity of pediatric disease, the likelihood of quicker recovery from illness and injury, differences in technique, and a higher prevalence of complications and death after tracheostomy in children. The duration of mechanical ventilation in children is often commensurate with the duration of the primary disease process with recovery. Earlier tracheostomies are preferred because of their multiple advantages. A recent meta-analysis reports that ET results in a shorter ICU stay, a larger number of ventilator-free days, and lower long-term mortality rates [27]. Pizza et al., in their retrospective analysis of children undergoing tracheostomy, showed a significant decrease in the incidence of ventilator-associated pneumonia with an ET (P=0.004, OR=0.39) [28]. Based on our results, we further provide evidence of an ET resulting in a shorter length of PICU stay, reduced ventilator-associated pneumonia incidence, and more ventilator-free days. Decannulation is always the target for any surgeon performing a tracheostomy and is tailored to the individual patient. Decannulation rates are reported as 15% to 80% [29]. These large variations in rates can be attributed to the changing indications for tracheostomy. Clinical readiness for decannulation usually takes three to six months on average, depending on the clinical indication for tracheostomy. 27/48 (56%) of our patients were decannulated during the study period. These are better than the decannulation rates (48%) reported by a study from Pakistan in 2010 [3]. Future directives at our institution include increasing decannulation rates through the implementation of a formal decannulation protocol. We recommended that a decannulation protocol should be instituted under the 2013 consensus statement guidelines and criteria from the American Academy of Otolaryngology (AAO) [30]. Decannulation at subsequent follow-ups for our patients was done within the next eight weeks to six months, with patients with upper airway obstruction being decannulated the earliest (within two months) and those with traumatic brain injury/underlying neurological conditions being decannulated after an average of six months. A standard decannulation protocol should be made available at all institutions offering a tracheostomy. In this article, we comprehensively review the indications, clinical characteristics, and outcomes of elective tracheostomies in critically ill children at our institution. We found that an ET in children requiring PMV is associated with better patient outcomes. Our study limitations included retrospective data collection from a single-center, small sample size. We suggest a multi-institutional analysis of care coordination, transitions for pediatric tracheostomy patients, and testing the effectiveness of interventions such as multidisciplinary tracheostomy care teams, standardized tracheostomy protocols and policies, staff education, and family involvement. We believe that these factors might eventually help improve the quality of care in these patients. We also suggest a detailed follow-up of decannulated patients.

## Conclusions

Tracheostomy in children is a relatively frequent procedure at our hospital within the critical care setting. PMV was the main indication for a tracheostomy. Earlier tracheostomy and a standardized protocolized approach are associated with better patient outcomes and reduced morbidity.
